# Deposition Mechanism and Properties of Plasma-Enhanced Atomic Layer Deposited Gallium Nitride Films with Different Substrate Temperatures

**DOI:** 10.3390/molecules27238123

**Published:** 2022-11-22

**Authors:** Fang-Bin Ren, Shi-Cong Jiang, Chia-Hsun Hsu, Xiao-Ying Zhang, Peng Gao, Wan-Yu Wu, Yi-Jui Chiu, Shui-Yang Lien, Wen-Zhang Zhu

**Affiliations:** 1 Xiamen Key Laboratory of Development and Application for Advanced Semiconductor Coating Technology, School of Opto-Electronic and Communication Engineering, Xiamen University of Technology, Xiamen 361024, China; 2 Fujian Provincial Key Laboratory of Nanomaterials, Fujian Institute of Research on the Structure of Matter, Chinese Academy of Sciences, Fuzhou 350002, China; 3 Department of Materials Science and Engineering, National United University, Miaoli 36063, Taiwan; 4 School of Mechanical and Automotive Engineering, Xiamen University of Technology, Xiamen 361024, China; 5 Department of Materials Science and Engineering, Da-Yeh University, Changhua 51591, Taiwan

**Keywords:** gallium nitride, plasma-enhanced atomic layer deposition, substrate temperature

## Abstract

Gallium nitride (GaN) is a wide bandgap semiconductor with remarkable chemical and thermal stability, making it a competitive candidate for a variety of optoelectronic applications. In this study, GaN films are grown using a plasma-enhanced atomic layer deposition (PEALD) with trimethylgallium (TMG) and NH_3_ plasma. The effect of substrate temperature on growth mechanism and properties of the PEALD GaN films is systematically studied. The experimental results show that the self-limiting surface chemical reactions occur in the substrate temperature range of 250–350 °C. The substrate temperature strongly affects the crystalline structure, which is nearly amorphous at below 250 °C, with (100) as the major phase at below 400 °C, and (002) dominated at higher temperatures. The X-ray photoelectron spectroscopy spectra reveals the unintentional oxygen incorporation into the films in the forms of Ga_2_O_3_ and Ga-OH. The amount of Ga-O component decreases, whereas the Ga-Ga component rapidly increases at 400 and 450 °C, due to the decomposition of TMG. The substrate temperature of 350 °C with the highest amount of Ga-N bonds is, therefore, considered the optimum substrate temperature. This study is helpful for improving the quality of PEALD GaN films.

## 1. Introduction

Gallium nitride (GaN), as a Group III nitride semiconductor material, has been widely used for optoelectronic device applications, such as light emitting diodes [1,2,3], high electron mobility transistors [4,5], semiconductor lasers [6,7], solar cells [8,9], and ultraviolet detectors [10,11], due to its advantages of wide direct band gap, high carrier concentration, high breakdown field strength, and excellent chemical durability [12]. GaN thin films can be grown using pulsed laser deposition [13,14], magnetron sputter epitaxy [15,16], molecular beam epitaxy [17,18], metal-organic chemical vapor deposition [19,20], and atomic layer deposition (ALD) [21,22]. Among them, ALD is an attractive technique for preparing high-quality films, owing to its unique self-limiting growth that allows for the deposition of highly conformal, pinhole-free thin films and precise thickness control at atomic level [23,24].

Recently, ALD GaN thin films have been developed by using diverse precursor sources. The gallium sources can be GaCl_3_, trimethylgallium (TMG), and triethylgallium (TEG), while the most common nitride source is NH_3_. The substrate temperature is one of the most crucial parameters in ALD growth; however, there are few studies investigating the effect of substrate temperature on the film properties. Kim et al. [25] prepared GaN films by thermal ALD with GaCl_3_ and NH_3_. They found that the substrate temperature window was between 500 and 750 °C, with a growth per cycle (GPC) of ~2.0 Å/cycle. Ozgit et al. [26] reported the self-limiting growth of GaN thin films via plasma-enhanced ALD (PEALD) with TMG and NH_3_ at a significantly lower substrate temperature range of 185-385°C. Shih et al. [27] reported the properties of GaN thin films grown by remote plasma ALD using TEG and NH_3_/H_2_ plasma in the temperature range of 200 to 500 °C. Alevli et al. [28] reported the effect of the substrate temperature (200–450 °C) on the structural and optical properties of hollow cathode plasma-assisted ALD GaN films with the use of TEG and N_2_+H_2_ plasma. Nevertheless, TMG is the most cost-effective Ga precursor, and the substrate temperature effect on the deposition mechanisms and properties of TMG+NH_3_-based PEALD GaN has not been fully investigated.

In this work, GaN thin films are prepared using PEALD using TMG and NH_3_. The substrate temperature varies from 200–450 °C, and its effect on the deposition mechanism and optical and structural properties is systematically studied.

## 2. Materials and Methods

Two-inch sapphire and four-inch silicon were used as substrates. The sapphire substrates were cleaned with deionized water, ethanol, isopropyl alcohol, and deionized water for 15 min in sequence, respectively. The sapphire substrates were then blow-dried by N_2_ and transferred to an oven at 80 °C for more than 20 min. The Si substrates were cleaned using a standard Radio Corporation of American cleaning process and then dipped in 2% hydrogen fluoride solution for 1 min to remove the surface native oxide. The wafers were then rinsed with deionized water and, finally, blow-dried in N_2_. GaN films were prepared by a PEALD system (R-200, Picosun, Espoo, Finland) using trimethylgallium (TMG, Aimou Yuan, Nanjing, China) as the gallium precursor. The TMG bubbler was maintained at a temperature of 0 °C to provide sufficient vapor pressure. High-purity (99.999%) nitrogen gas was used not only as the purging gas but also the carrier gas to deliver TMG vapors from the bubbler to deposition chamber. The plasma was produced in a microwave cavity using inductive coupling of RF power (Litmas RPS, Advanced Energy, Denver, CO, USA) with the gas mixture of NH_3_ and Ar. The substrate temperature varied from 200 to 450 °C. The time sequence for one PEALD cycle was: TMGs pulse time (0.1 s), N_2_ purge time (4 s), NH_3_ plasma (13 s), N_2_ purge time (6 s). These pulse times and purge times were the optimized values. A total of 1000 cycles were performed for each sample. The detailed PEALD parameters are summarized in Table 1.

The thickness and refractive indices of the GaN films were measured on silicon wafers by a spectroscopic ellipsometer (SE, SENTECH SE 800 DUV, Berlin, Germany). The optical transmittance measurements of the films were performed on the sapphire substrates by a UV–Vis spectrophotometer (Lambda850, PerkinElmer, MA, USA) at room temperature. The species in plasma during the deposition process were characterized using an optical emission spectrometer (OES, SD2048DL, Verity, Carrollton, TX, USA). The crystalline structure of the films was investigated by grazing incidence X-ray diffraction (GIXRD, Rigaku TTRAX III, Ibaraki, Japan) with an X-ray wavelength of 0.154 nm and an incident angle of 1°. The morphology of the film surface was observed by a field emission scanning electron microscopy (FESEM, Sigma 500, Zeiss, Germany) and atomic force microscopy (AFM, Bruker, MA, USA). The chemical states were analyzed by using X-ray photoelectron spectroscopy (XPS, ESCALAB 250Xi, Thermo Fisher, Waltham, MA, USA). The film surface was pre-sputtered by an Ar ion beam for 0.5 min before recording of the XPS spectra to exclude the influence of surface contamination. The binding energies of the XPS peaks were calibrated using the C1s peak (284.8 eV) as a standard reference. Secondary ion mass spectroscopy (SIMS, TOF-SMIS V, ION-TOF, Münster, Germany) measurements were carried out to acquire the depth profile of elemental distribution. The photoluminescence spectra of the GaN films were obtained using a fluorescence spectrometer (FLS 980, Edinburgh Instruments, Edinburgh, UK) with an excitation wavelength of 325 nm at room temperature.

## 3. Results and Discussion

**Figure 1:**
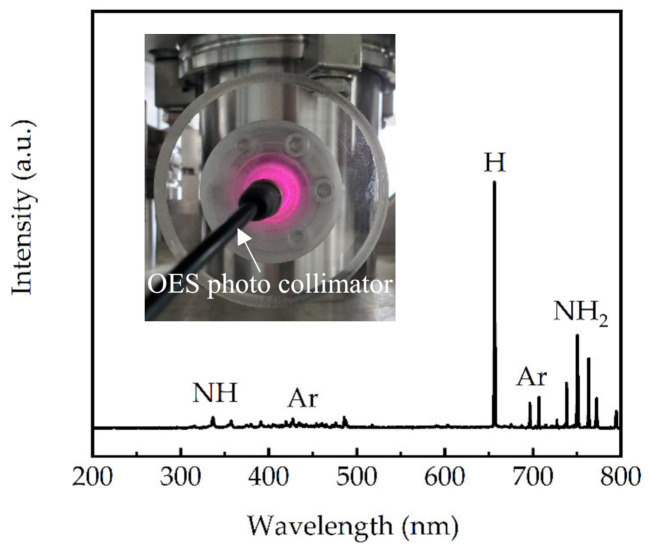
OES measurement and spectrum of the NH_3_+Ar plasma at 2500 W.

Figure 1 shows the OES measurement and spectrum of the NH_3_+Ar plasma. The signals corresponding to NH, Ar, H, and NH_2_ were identified according to the literature [29,30]. The intensive H signal indicated the enormous dissociation of NH_3_ into NH_2_ and NH. Consequently, the initial stage of the deposition process could be reasonably expected to be NH_x_ ligands (x = 1,2) adsorbed on the substrate surface [31]. When the TMG was supplied, the first-half of the ALD reaction could be described by the following equation:

Ŝ-NH_x_ + Ga(CH_3_)_3_ →Ŝ-N-[Ga(CH_3_)_2_]_x_ + CH_4_, (1)

where Ŝ represents the substrate surface. The abstract of H from NH_x_ ligands formed Ga-N bonds, accompanied by the release of CH_4_ volatile byproducts. It should be noted that the NH surface ligands were not as ideal as NH_2_ from the perspective of the ALD ligand exchange process, as they had unsaturated bonds, which may be filled in next few cycles or eventually remain as a dangling bond in the film. Fortunately, from the OES spectrum, the amount of NH species was much smaller than NH_2_. The second-half reaction involved nitridation and was given by:

Ŝ-N-Ga(CH_3_) + (NH+NH_2_+H)_plasma_ → Ŝ-N-Ga-NH_x_ + CH_4_, (2)

The surface ligands returned to the NH or NH_2_ state. The first- and second-half reactions governed a complete PEALD cycle, which was repeated until reaching the required GaN film thickness. This growth model was analogous to the PEALD AlN using TMA, which was structurally similar to TMG [32]. As a result, the substrate temperature was expected to affect the physisorption, chemisorption of the precursors, and chemical reaction rate of the ligand exchange process.

**Figure 2:**
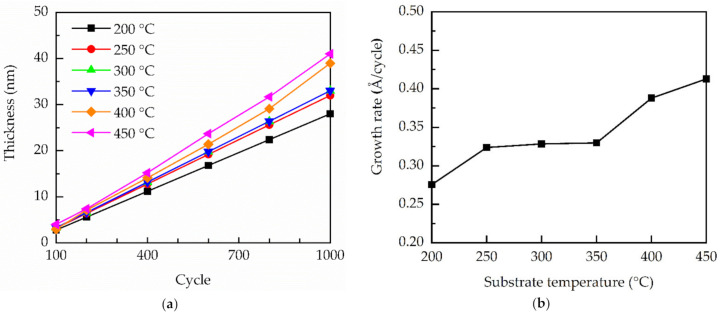
(**a**) Thickness of GaN films versus ALD cycle number for different substrate temperatures; (**b**) GPC of GaN films with different substrate temperatures.

The thickness of GaN films prepared at different substrate temperatures as a function of PEALD cycle number is depicted in Figure 2a. At substrate temperatures less than 400 °C, the film thickness increased linearly with the PEALD cycle, which was a good sign of ALD growth mode [33]. The linear relationship becomes weaker at the substrate temperatures of 400 and 450 °C, implying a deviation from ALD growth. The GPC of the films in Figure 2b is defined as the thickness divided by the total ALD cycles. The GPC at 200 °C was as low as the 0.28 Å/cycle, which was a consequence of the relatively weak chemisorption and chemical reaction rates. The GPC increased to 0.32 Å/cycle, and a clear PEALD temperature window with a nearly constant GPC was demonstrated in 250–350 °C. Further increasing the substrate temperature to 400 or 450 °C led to an increase in GPC, ascribed to the partial decomposition of the TMG [21]. In this case, TMG tended to decompose into smaller units, such as dimethylgallium (Ga(CH_3_)_2_) or monomethyl-gallium (GaCH_3_), and their further decomposition led to the formation of clusters of Ga [21]. Some CVD-like reactions occurred, and self-limiting saturation surface reactions were not applicable in the vicinity of the decomposed TMG. As a result, the ALD and CVD-like growth modes coexisted at high substrate temperatures. Since the CVD growth rate of the GaN films was typically much higher than the ALD growth rate, increasing the temperature to 400 or 450 °C led to an increase in GPC. In the present work, the maximum substrate temperature was 450 °C, due to the limitation of the used PEALD system. Nevertheless, this study focused on the characteristics of PEALD GaN films. The GPC versus substrate temperature revealed that the ALD temperature window was in the range of 200 to 350 °C. The substrate temperatures of 400 and 450 °C already led to CVD-like reactions. Therefore, a higher substrate temperature (>450 °C) was not considered, since the films would be grown in a more deviated ALD mode.

**Figure 3:**
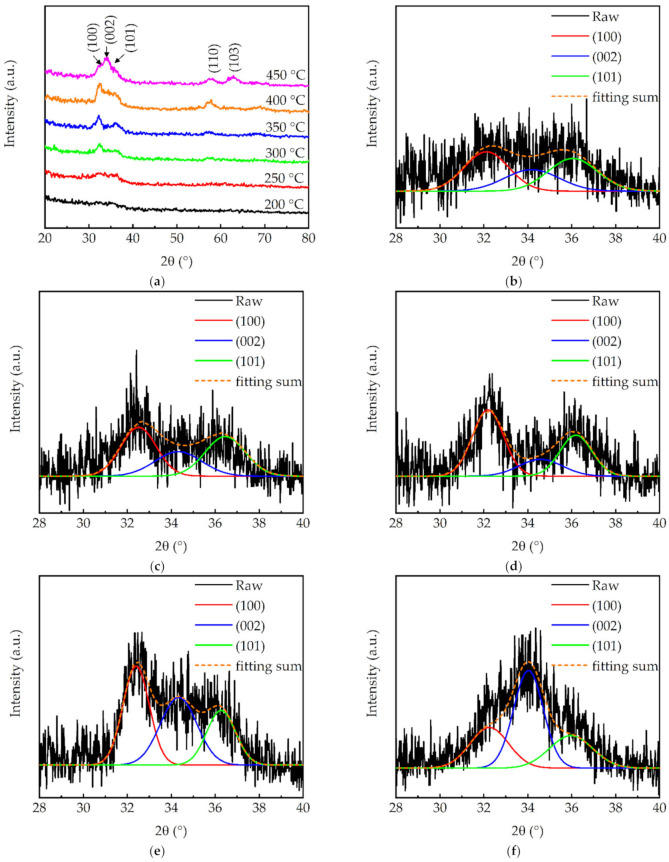

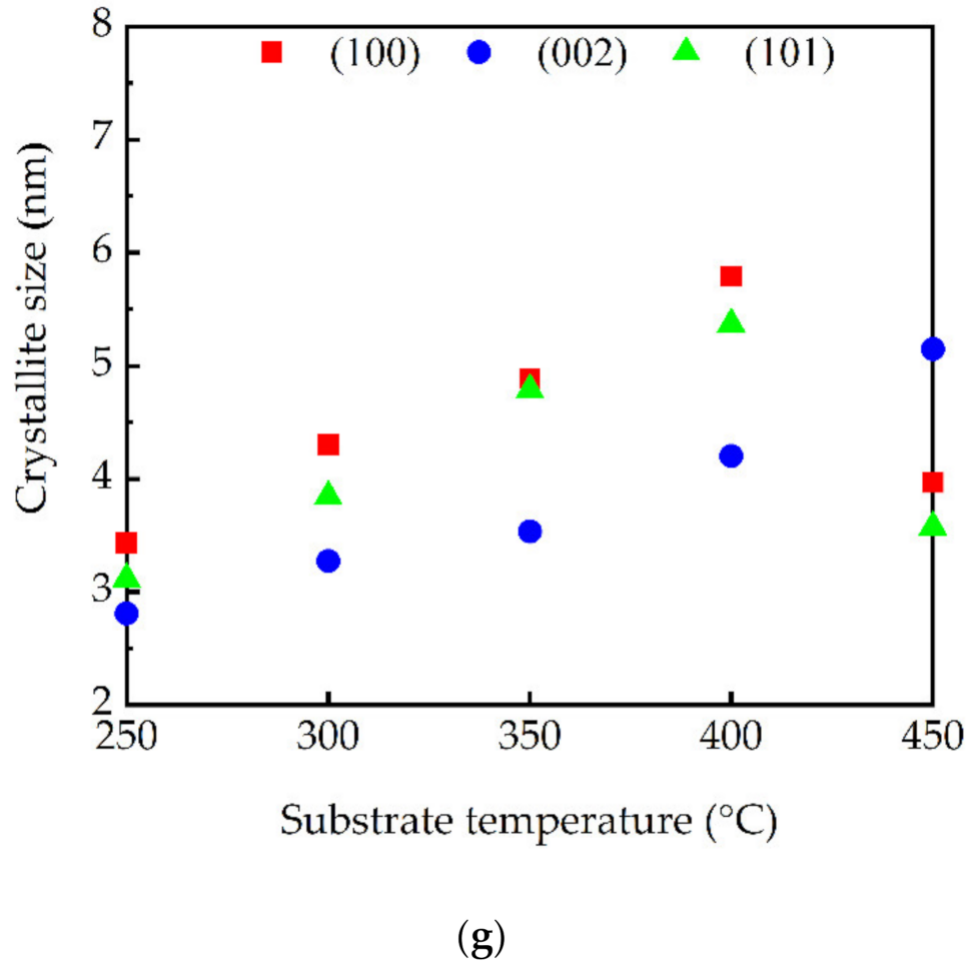
(**a**) GIXRD patterns of the GaN films with different substrate temperatures. The spectra have been vertically shifted for clarity. Peak deconvolution for the films prepared at (**b**) 250, (**c**) 300, (**d**) 350, (**e**) 400, and (**f**) 450 °C. (**g**) Crystallite size for GaN (100), (002), and (101) planes.

Figure 3a shows the GIXRD patterns for the PEALD GaN films prepared at different substrate temperatures. There were diffraction peaks at 32.4°, 34.6°, 36.9°, and 57.8° corresponding to the (100), (002), (101), and (110) orientations of the GaN wurtzite structure, respectively, according to the standard PDF card (JCPDS# #50-0792). Figure 3b–f show the peak fitting of (100), (002), and (101) phases. The peak intensities were very low at 200 °C, indicating that the film should have been nearly amorphous. At 250 °C, the crystalline orientations were mainly (100) and (101) with similar peak areas. At 300–350 °C, the (100) phase developed faster than others, whereas the (002) phase increased rapidly at 400 °C and eventually became the leading phase when the substrate temperature reached 450 °C. As the (002) plane posed the lowest surface energy, the GIXRD result indicated that, at low temperatures of less than 400 °C, the Ga and N atoms did not have sufficient energy and could only move a short distance around the position where they were adsorbed. A high enough substrate temperature (in this case >400 °C) can facilitate the migration of Ga and N to the most energetically favorable (002) orientation. Furthermore, Figure 3g shows the average crystallite size (D) for the GaN (100), (002) and (101) planes, obtained using Scherrer’s equation:

(3) $$D=\frac{k\lambda }{\beta \cos\theta }$$

where λ is the X-ray wavelength (0.15405 nm), β is the full width at half maximum of diffraction peaks, and θ is the Bragg’s diffraction angle. The average crystallite sizes for the (100), (101), and (002) planes increased when the substrate temperature increased from 250 to 400 °C. The crystallite sizes for the (100) and (101) planes significantly decreased at 450 °C, whereas the crystallite size for the (002) plane further increased. This indicates a change in crystalline structure from (100) and (101) to (002)-dominated. It should be noted that the weak GaN GIXRD peaks in this work may be ascribed to the high oxygen content in the films (as latter shown in the XPS result). In the study of Shih et al. [27], the PEALD GaN films had similar GIXRD peak intensities and oxygen incorporation. Some methods for improving the crystallization quality of GaN films have been proposed. Liu et al. [34] reported the growth of single-crystalline GaN epilayers by using a baking and plasma pretreatment of the substrate prior to the GaN plasma-enhanced atomic layer deposition. Lee et al. [35] achieved high-quality GaN epilayers using atomic layer annealing and epitaxy, in which a low power He/Ar plasma treatment was introduced in each cycle of atomic layer deposition, resulting in a significant improvement of the GaN crystal quality. These methods can be used to improve the crystalline quality of GaN films in our future studies.

**Figure 4:**
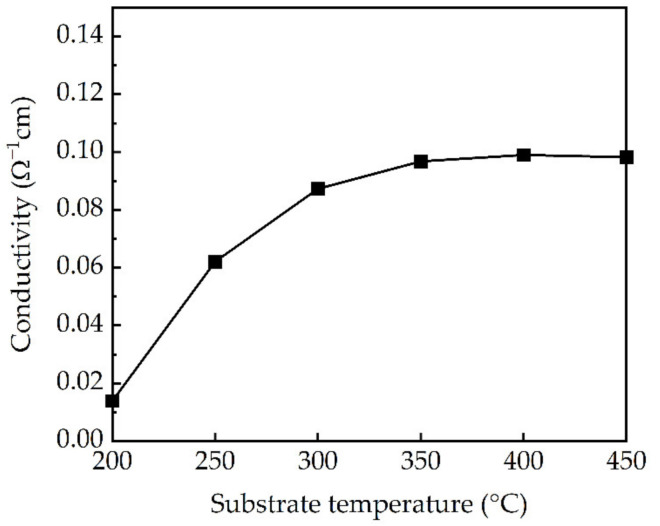
Conductivity of PEALD GaN films deposited at different substrate temperatures.

Figure 4 shows the conductivity of the PEALD GaN films obtained by four-point probe measurements. It found that increasing the deposition temperature led to an increase in conductivity, due to the improved crystalline structure (as shown in GIXRD results) that has less charge carrier scatterings [36].

**Figure 5:**
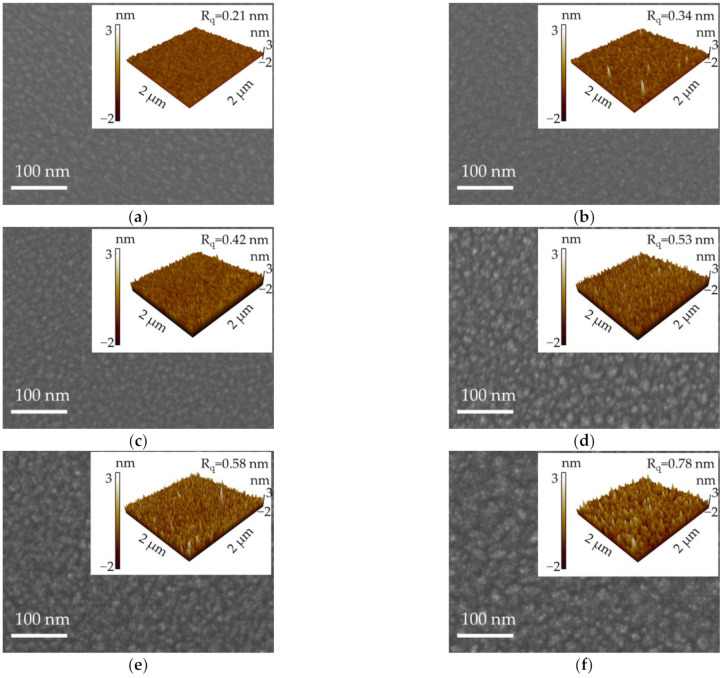
SEM images for the PEALD GaN films deposited at (**a**) 200, (**b**) 250, (**c**) 300, (**d**) 350, (**e**) 400, and (**f**) 450 °C. The insets were the corresponding AFM images.

The topographical images of the PEALD GaN films prepared at different temperatures are shown in Figure 5. It can be seen that a flat and smooth surface was acquired at the substrate temperatures of 200, 250, and 300 °C, as shown in Figure 5a–c. When the substrate temperature rose from 350 to 450 °C, the grains became larger, as seen in Figure 5d–f. Moreover, the insets were the AFM images of the films, showing an increased R_q_ from 0.21 to 0.78 nm with the increase in substrate temperature. Alevli et al. [28] used PEALD to grow GaN films at 200 and 450 °C, and the films had root-mean-square surface roughness values of 0.36 and 0.98 nm, respectively, which were similar to our experimental results. They explained that the increased surface roughness with increasing substrate temperature was due to different growth rates along different crystallographic directions [28,36]. Furthermore, it was reported that the surface roughness of PEALD GaN films would increase with the film thickness [36,37]. According to the GPC results in this work, the GaN film was thicker when deposited at a higher substrate temperature as the total PEALD cycle was fixed. This is another possible reason for the increased surface roughness.

**Figure 6:**
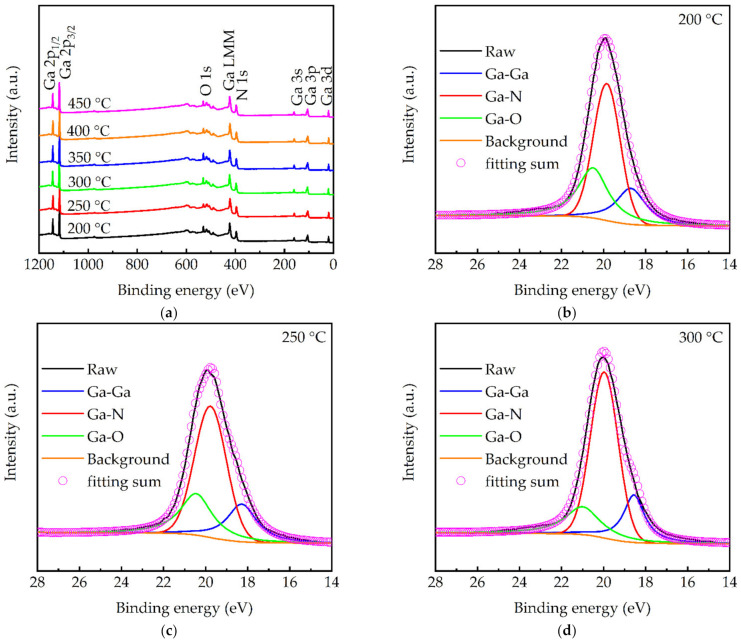

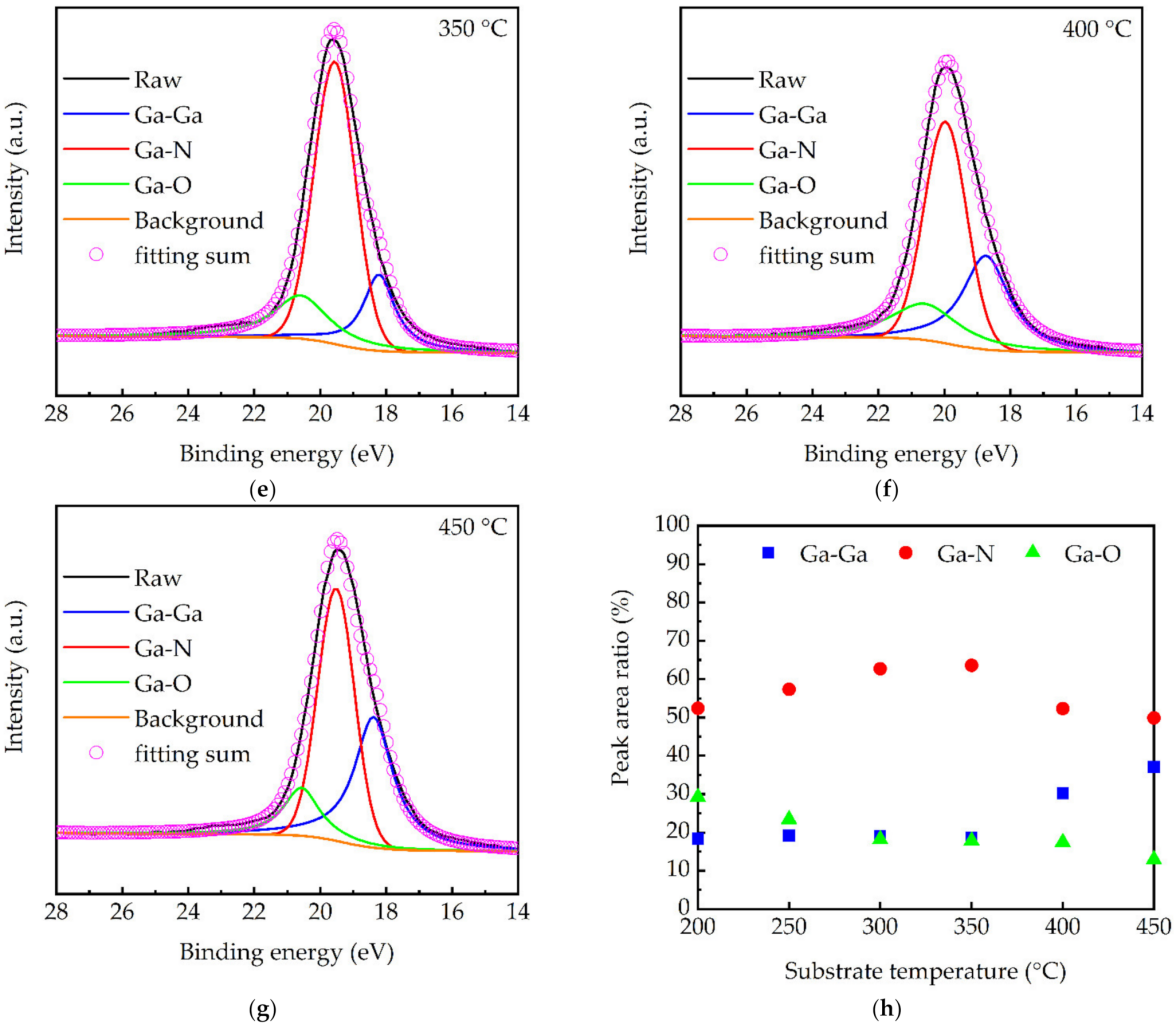
(**a**) XPS survey spectra of the GaN films deposited at different substrate temperatures. The spectra have been vertically shifted for clarity. Ga 3d core level XPS spectra for (**b**) 200, (**c**) 250, (**d**) 300, (**e**) 350, (**f**) 400, and (**g**) 450 °C. (**h**) Ga-Ga, Ga-N, and Ga-O peak area ratios, as a function of substrate temperature.

**Figure 7:**
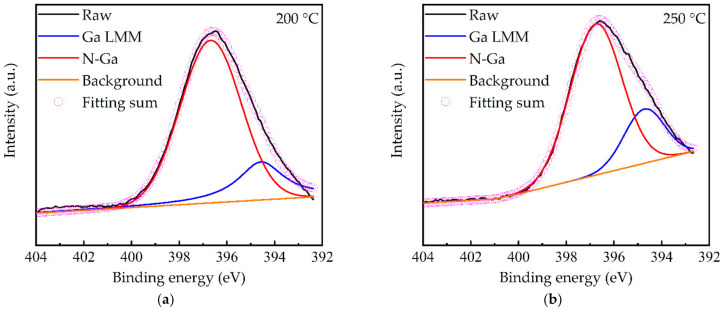

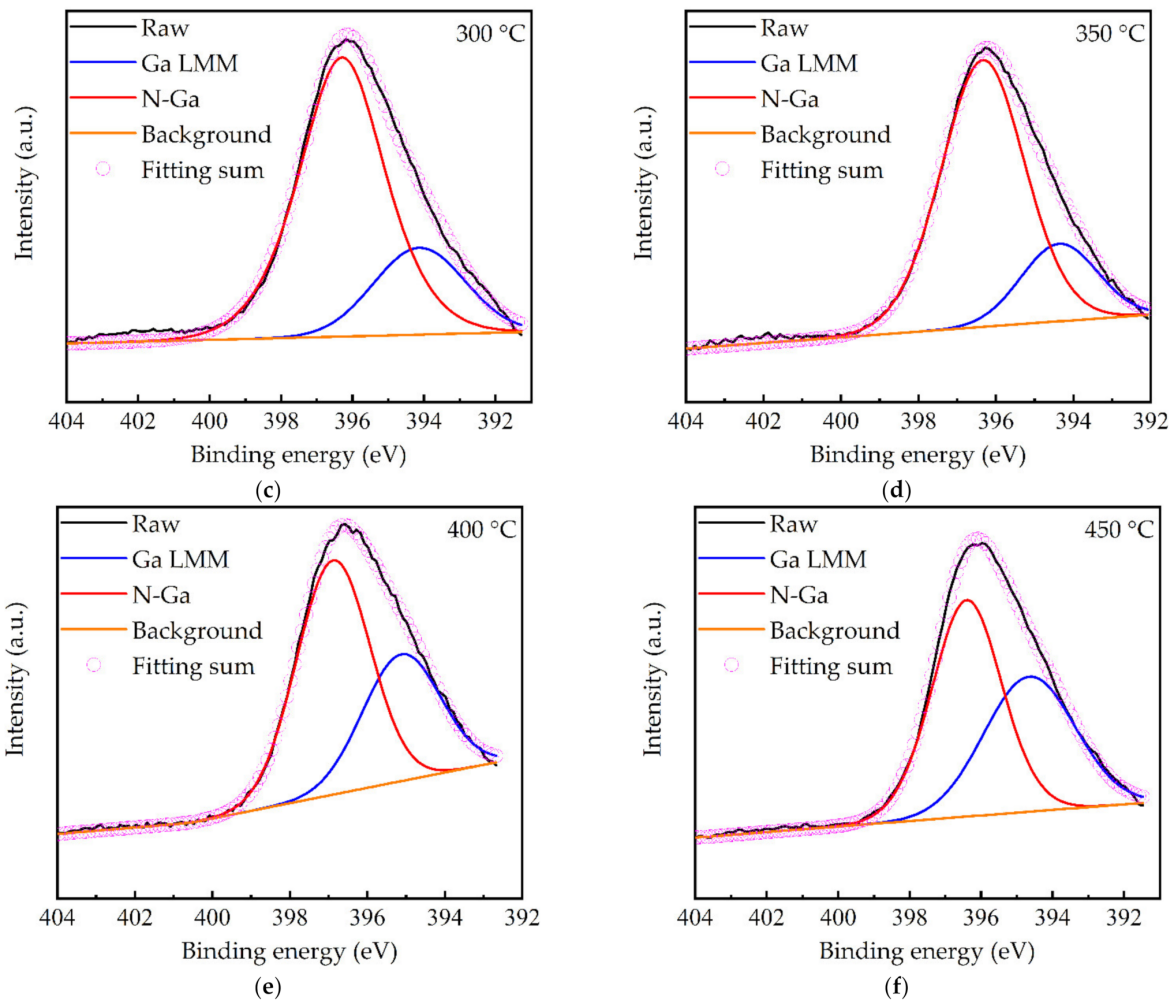
N 1s core level XPS spectra for (**a**) 200, (**b**) 250, (**c**) 300, (**d**) 350, (**e**) 400, and (**f**) 450 °C.

**Figure 8:**
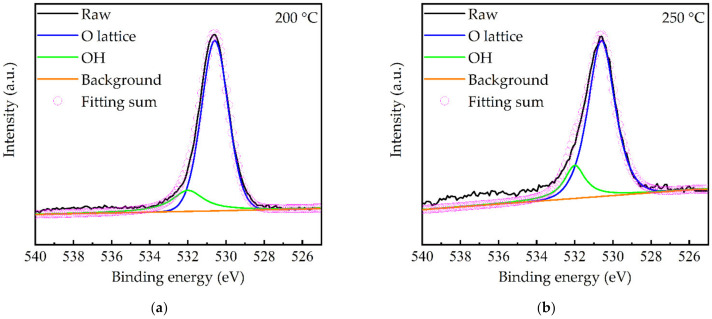

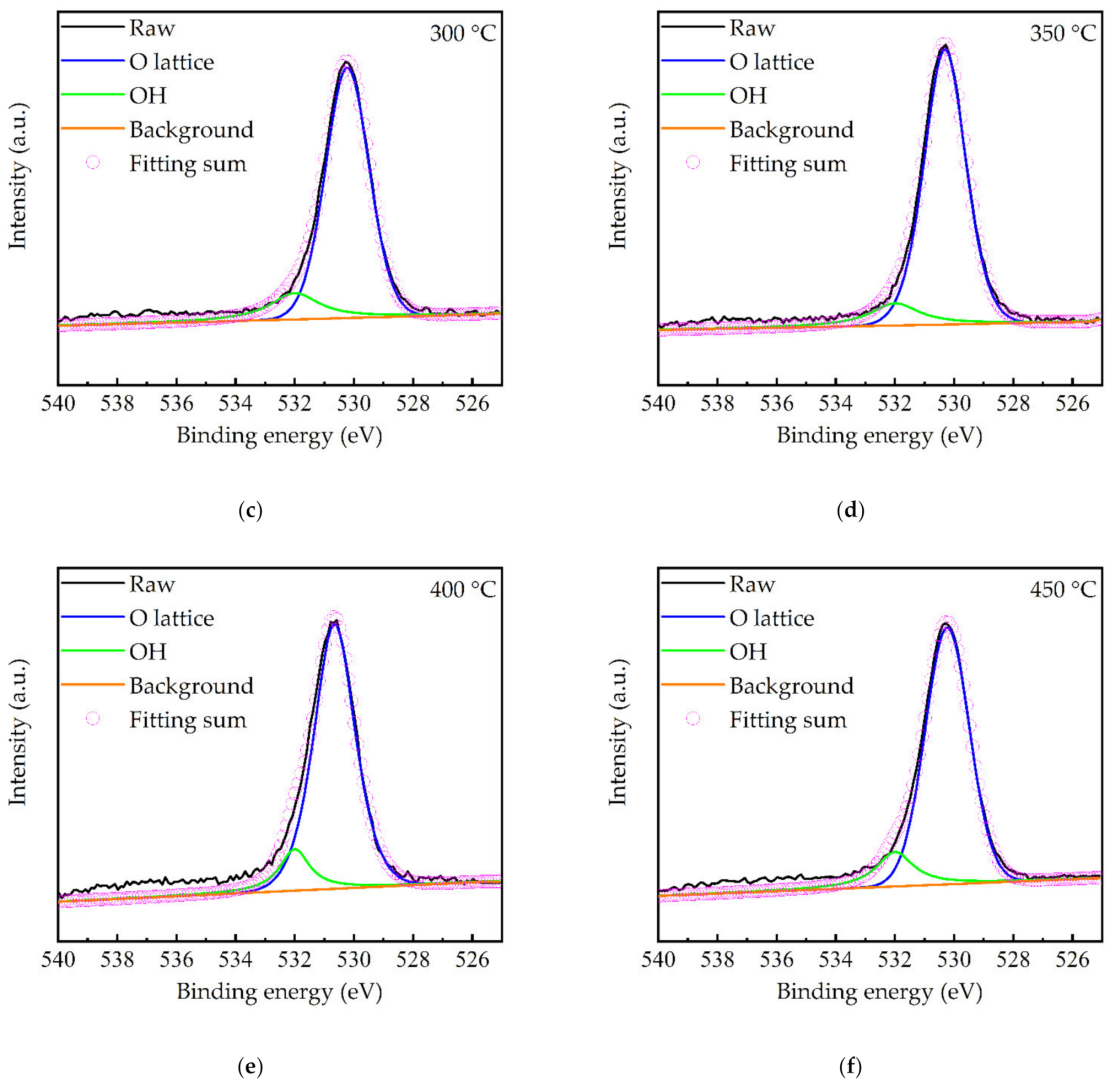
O 1s XPS spectra for the PEALD GaN films deposited at (**a**) 200, (**b**) 250, (**c**) 300, (**d**) 350, (**e**) 400, and (**f**) 450 °C.

Figure 6a shows the XPS survey spectra of the GaN films prepared at different substrate temperatures. The Ga and N peaks were observed and assigned according to the literature [28,38]. The unexpected oxygen incorporation was also reported in other PEALD GaN studies. For instance, Ozgit et al. [39] reported that the most possible origin of the oxygen contamination in GaN was the quartz tube of the induced coupled plasma source. Another reason in this work was due to the residual oxygen in the deposition chamber, since the used PEALD system could only reach a vacuum level of 10^−4^ Torr. The C peak is almost absent, indicating the complete nitridation in Equation (2). The Ga 3d peaks for different substrate temperatures were further decomposed, as shown in Figure 6b–g. The subpeaks at 18–19.2 eV, 19.4–20.2 eV, and 20.3–20.9 eV were assigned to Ga-Ga, Ga-N, and Ga-O, respectively [40,41,42]. The area ratios of these subpeaks were summarized in Figure 6h. The Ga-N peak area ratio increased with the increased substrate temperature and reached its maximum at 350 °C. The increased Ga-N peak area ratio stemmed from the decreased Ga-O component, presumably due to the enhanced crystalline structure, which was more resistant to the diffusion of residual oxygen into the films. Furthermore, from the N 1s spectra in Figure 7, no N-O peak can be found. From the O 1s spectra shown in Figure 8a–f, a higher binding energy peak at 532 eV can be seen, corresponding to OH and a lower binding energy at 530.8 eV assigned to lattice oxygen [42]. It is, thus, deduced that the oxygen is incorporated in the forms of Ga_2_O_3_ and Ga-OH. The elemental composition in Table 2 also supports the decreased oxygen at increasing substrate temperatures. The drop of Ga-N peak ratio after 350 °C is attributed to the increased Ga-Ga metal component. This can be explained by the partially decomposed TMG into Ga (as well as CH_3_) fragments that resulted in extra Ga-Ga bonds. Although not demonstrated in this work, PEALD GaN films have a wide range of applications, despite the presence of oxygen in the films. For instance, Qiu et al. [43] deposited a compact and uniform PEALD GaN thin layer, with an oxygen content of ~20 at.%, as an electron transport layer or a buffer layer for planar perovskite solar cells. The cell efficiency increased from 10.38% without GaN buffer layer to 15.18%. He et al. [22] directly deposited the PEALD GaN thin films, with an oxygen content of 11.61 at.%, on multilayer graphene in order to improve the performance of GaN-based optical and high-power devices. Tekcan et al. [44] prepared PEALD GaN films at 200 °C and demonstrated the viability of PEALD GaN-based ultraviolet photodetectors with temperature-sensitive substrates used in flexible and transparent optoelectronic devices.

**Figure 9:**
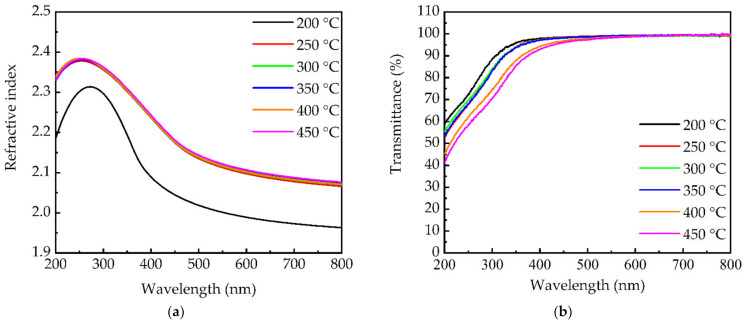

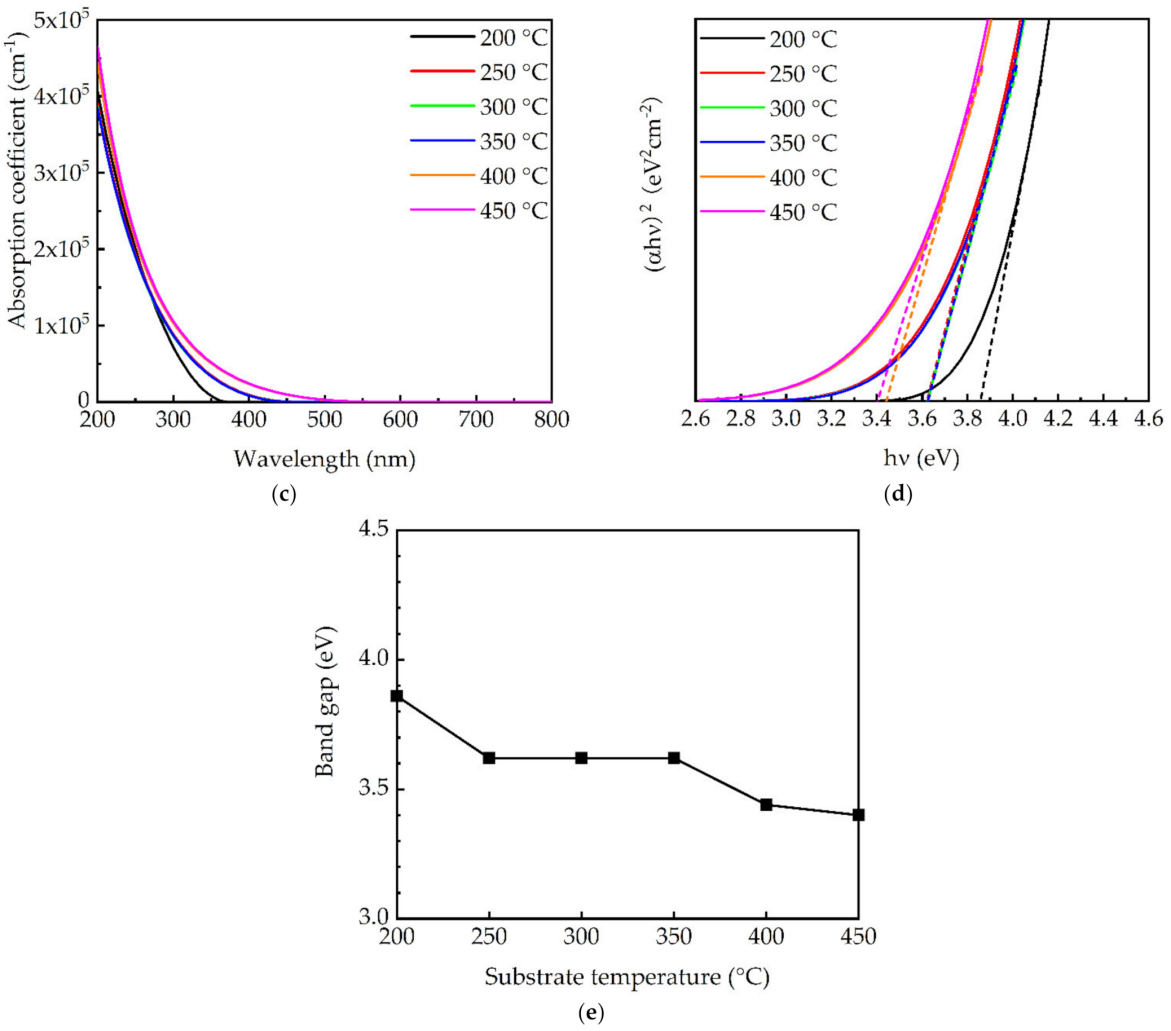
(**a**) Refractive index spectra, (**b**) transmittance spectra, (**c**) absorption coefficient spectra, (**d**) curves of (αhν)^2^ versus hν, and (**e**) band gap for GaN films prepared at different substrate temperatures.

The optical properties of the PEALD GaN films at different substrate temperatures were investigated. Figure 9a shows the refractive indices of the films. In general, the refractive index is related to the film compactness. Compared to the 200 °C-prepared sample, the samples prepared in the ALD process window (250–350 °C) had a higher refractive index, due to the higher compactness. The films deposited at 400 and 450 °C showed a comparable refractive index, due to the enhanced crystalline structure that compensates the effects of the CVD-like (or deviated ALD) growth mode. Figure 9b,c shows the transmittance and absorption coefficient spectra of the films, respectively. It can be seen that the curves have a red-shift absorption edge at increasing substrate temperature, indicating a reduced optical band gap. To confirm this, the band gaps of the films were evaluated using the Tauc plot method [45]. In Figure 9d, the plot of (αhν)^2^ versus hν is illustrated for each substrate temperature, with α being the absorption coefficient and hν being the photon energy. By extrapolating the linear region of the curves to the hν-axis, the interception gave the band gap of the films. The extracted band gap value in Figure 9e decreases from 3.86 to 3.4 eV, when the substrate temperature increases from 200 to 450 °C. Considering that the Ga_2_O_3_ had a band gap higher than GaN, the higher band gap at 200 °C was attributed to the higher amount of the Ga-O component in the film. There was little drop in band gap when the substrate temperature increased from 250 to 350 °C. Further increasing the temperature caused a relatively obvious decrease in band gap, due to the combined effect of the reduced Ga-O and increased Ga-Ga components. In the literature, Qiu et al. [43] prepared 50–200 PEALD cycles of GaN films at 280–300 °C, and the band gap values of the films ranged from 3.95 to 3.58 eV. Alevli et al. [28] used hollow cathode PEALD to grow GaN films at 200 and 450 °C, and the corresponding band gap values were 3.6 and 3.52 eV, respectively. Seda Kizir et al. [46] prepared GaN thin films on sapphire substrates via hollow-cathode PEALD at 200 °C, and the bandgap was around 3.61 eV. Overall, the band gap values for the PEALD GaN films in this study were in the range of the reported values.

**Figure 10:**
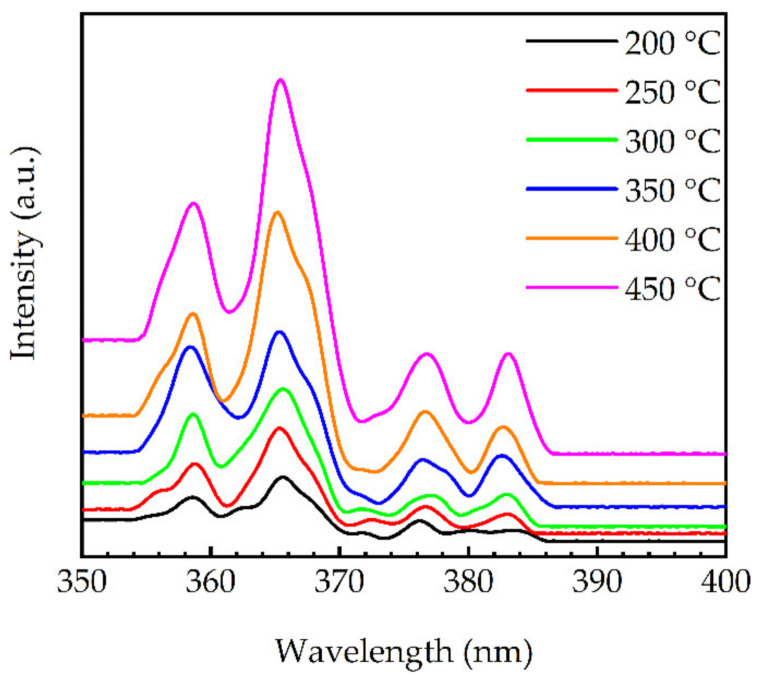
Photoluminescence spectra for the PEALD-GaN films deposited at different substrate temperatures. The spectra have been vertically shifted for clarity.

The photoluminescence spectra of the PEALD-GaN films deposited at different temperatures are shown in Figure 10. The most intense peak was at 365 nm, which was close to the band edge of GaN and attributed to exciton recombination [47,48]. The free exciton peak at 358 nm was observed [49]. The peaks at around 388 nm were typically associated with structural defects [50,51]. It was also reported that oxygen incorporation led to shallow donor states in GaN, and the luminescence between electrons captured in the shallow donors and holes localized by the oxidized surface states could emit an energy below the GaN band gap [47,52]. This could be another explanation for the presence of the peaks at around 378 and 388 nm, as in this study, there was a significant oxygen content in the GaN films. It can also be seen that the peak intensities increased with the increased substrate temperatures.

**Figure 11:**
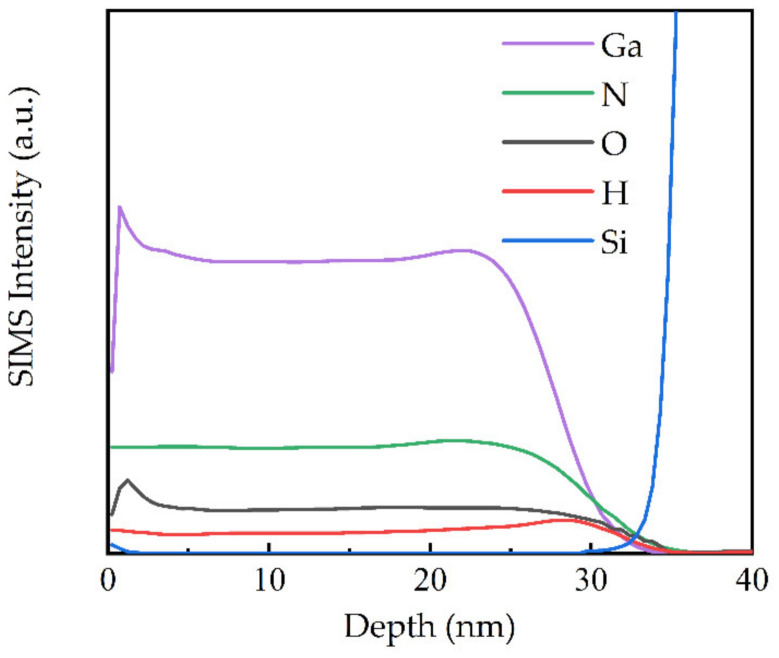
SIMS depth profiles of Ga, N, O, H, and Si intensities for the GaN film deposited at 350 °C.

The film deposited at 350 °C, which was grown in ALD mode and with the highest Ga-N bond ratio, was then considered to be the optimized substrate temperature. The SIMS profile of the 350 °C-prepared film is shown in Figure 11. Except for the surface layer (3 nm), which may be contaminated by the environment, the elemental depth distribution in the bulk region of the film was fairly uniform. The hydrogen signal was detected, which should originate from the NH_3_ plasma. The hydrogen atoms in the films could be in the form of N-H, C-H, or O-H. However, the XPS results show that the carbon peak was negligible, and the N 1s deconvolution only showed N-Ga and Ga Auger components. Therefore, from the higher binding energy OH component in the deconvoluted O 1s spectra, as discussed in Figure 8, it was deduced that hydrogen atoms were incorporated into the films, mainly in the form of Ga-OH.

## 4. Conclusions

PEALD GaN films were prepared with different substrate temperatures. The ALD self-limiting surface reactions can be achieved at substrate temperatures of 250–350 °C. The GIXRD result indicated that the GaN films undergo phase transition from nearly amorphous at 200 °C through (100) phase-dominated at 250–400 °C to (002) phase at 450 °C. At 350 °C, the GaN film showed the highest Ga-N proportion. Films deposited at lower substrate temperatures suffered from residual oxygen incorporation, while the higher substrate temperatures were helpful for reducing the oxygen content, but led to Ga-Ga formation, due to the TMG decomposition. All the films had a transmittance of greater than 90%, with a red-shift absorption edge indicating a reduced band gap at increasing substrate temperatures. This study will help in the promotion of the PEALD technique in GaN thin film preparation.

## Figures and Tables

**Table 1:** Deposition parameters for PEALD GaN thin films.

Parameter	Value
TMG bubbler temperature (°C)	0
Substrate temperature (°C)	200–450
TMG pulse time (s)	0.1
TMG purge time (s)	4
TMG carrier gas flow rate (sccm)	120
NH_3_ pulse time (s)	13
NH_3_ purge time (s)	6
NH_3_ flow rate (sccm)	30
NH_3_ plasma power (W)	2500
Ar flow rate (sccm)	160

**Table 2:** Ga, N, and O atomic ratios for the GaN films prepared at different substrate temperatures.

Substrate Temperature (°C)	Ga (at.%)	N (at.%)	O (at.%)
200	47.52	34.74	17.74
250	47.81	35.13	17.06
300	48.65	35.4	15.95
350	48.91	35.93	15.16
400	53.01	32.82	14.17
450	54.54	33.18	12.28

## Data Availability

Not applicable.

## Sample Availability

Samples of compounds are available from the authors.
